# SmeltCam: Underwater Video Codend for Trawled Nets with an Application to the Distribution of the Imperiled Delta Smelt

**DOI:** 10.1371/journal.pone.0067829

**Published:** 2013-07-04

**Authors:** Frederick Feyrer, Donald Portz, Darren Odum, Ken B. Newman, Ted Sommer, Dave Contreras, Randall Baxter, Steven B. Slater, Deanna Sereno, Erwin Van Nieuwenhuyse

**Affiliations:** 1 Bay Delta Office, Bureau of Reclamation, Sacramento, California, United States of America; 2 Technical Service Center, Bureau of Reclamation, Denver, Colorado, United States of America; 3 SureWorks LLC., Longmont, Colorado, United States of America; 4 U.S. Fish and Wildlife Service, Lodi, California, United States of America; 5 California Department of Water Resources, West Sacramento, California, United States of America; 6 California Department of Fish and Wildlife, Stockton, California, United States of America; 7 Contra Costa Water District, Concord, California, United States of America; The Australian National University, Australia

## Abstract

Studying rare and sensitive species is a challenge in conservation biology. The problem is exemplified by the case of the imperiled delta smelt *Hypomesus transpacificus*, a small delicate fish species endemic to the San Francisco Estuary, California. Persistent record-low levels of abundance and relatively high sensitivity to handling stress pose considerable challenges to studying delta smelt in the wild. To attempt to overcome these and other challenges we have developed the SmeltCam, an underwater video camera codend for trawled nets. The SmeltCam functions as an open-ended codend that automatically collects information on the number and species of fishes that pass freely through a trawled net without handling. We applied the SmeltCam to study the fine-scale distribution of juvenile delta smelt in the water column in the upper San Francisco Estuary. We learned that during flood tides delta smelt were relatively abundant throughout the water column and that during ebb tides delta smelt were significantly less abundant and occurred only in the lower half and sides of the water column. The results suggest that delta smelt manipulate their position in the water column to facilitate retention in favorable habitats. With the application of the SmeltCam we increased the survival of individual delta smelt by 72% compared to using a traditional codend, where all of the fish would have likely died due to handling stress. The SmeltCam improves upon similar previously developed silhouette photography or video recording devices and demonstrates how new technology can be developed to address important questions in conservation biology as well as lessen the negative effects associated with traditional sampling methods on imperiled species.

## Introduction

Conservation of endangered species faces many challenges. A key difficulty is studying and monitoring populations in which individuals are by definition low in abundance and rarely observed. Solutions to this problem often address sampling design and fitting appropriate models to data [1], [2]. Potential solutions could also involve alternative methods and new technology. For example, remote photography or video methods are commonly used in ecology to address a variety of research questions [3], [4], [5], [6] and could be applied to the study of imperiled species. In particular, underwater video systems are becoming increasingly popular for studying fishes in marine [7], [8], estuarine [9], [10], [11] and freshwater habitats [12], [13]. They are especially desirable when a key objective is to minimize or avoid the adverse effects of handling stress associated with traditional sampling methods [14], [15].

**Figure 1:**
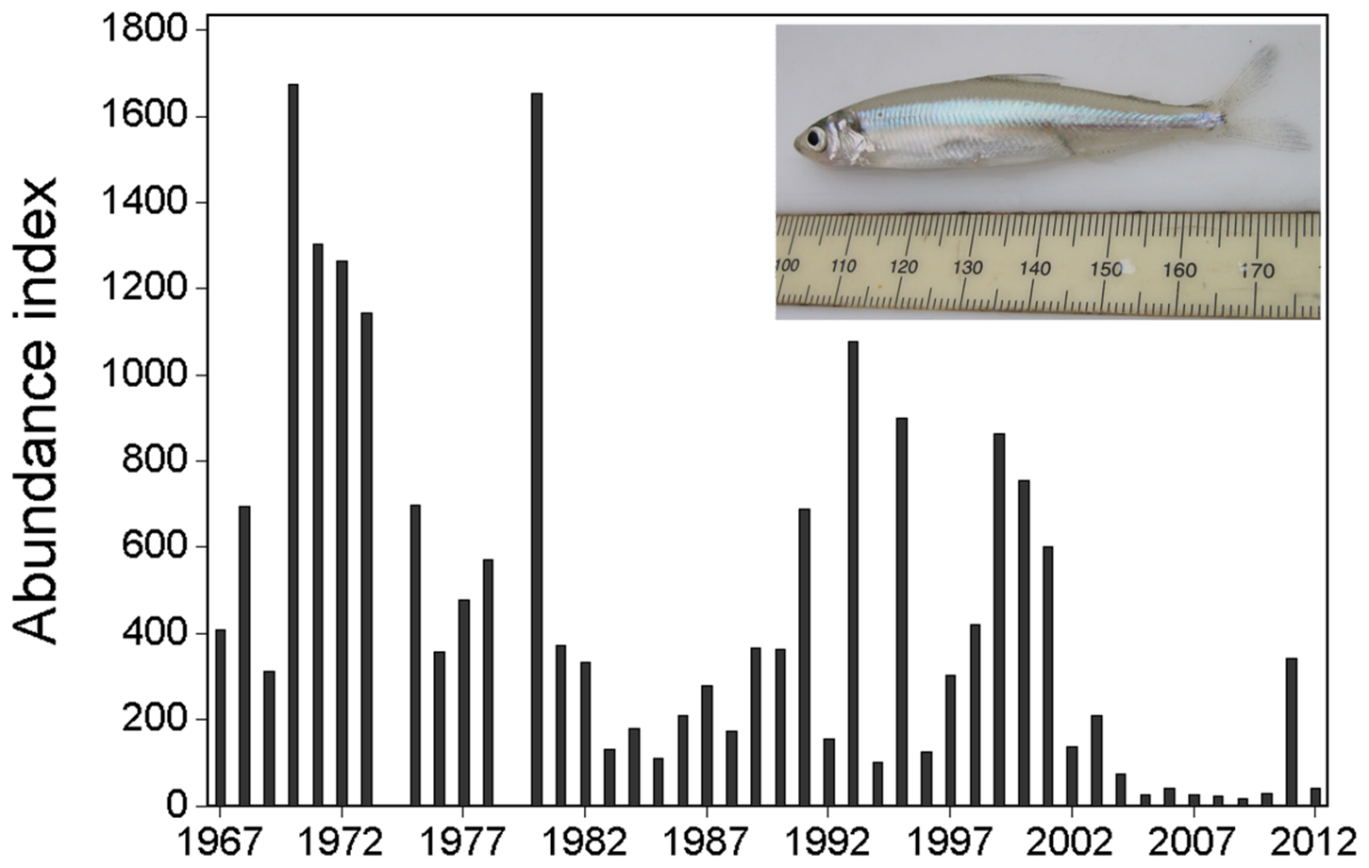
Time series of delta smelt abundance indices (unitless) from the California Department of Fish and Wildlife’s Fall Midwater Trawl Survey. No sampling was conducted in 1974 or 1979. Inset is a photograph of a delta smelt collected during the study. Rule increments are millimeters.

The need for alternative methods to study imperiled species is exemplified by the case of the delta smelt *Hypomesus transpacificus* in the upper San Francisco Estuary. The delta smelt is a formerly abundant euryhaline pelagic fish endemic to the upper San Francisco Estuary that has experienced substantial declines in abundance (Fig. 1) [16], [17]. Abundance declines have been attributed to multiple interacting factors including foodweb alterations, physical habitat loss, contaminants and water diversions [16], [17], [18], [19]. To complicate matters further, the delta smelt is a small (maximum fork length ∼ 90 mm) fish that typically dies with minimal handling stress (20); it is assumed that most individuals collected in routine monitoring surveys do not survive. Because of their small size and delicate nature, wild delta smelt have not been tagged for remote tracking or for mark-recapture studies with presently available tools or technology (but see [21] for a study on cultured delta smelt). Delta smelt live in turbid pelagic habitats [22], [23] and therefore cannot be directly observed in their natural environment. Currently available hydroacoustic methods are of limited utility because three other fish species (longfin smelt *Spirinchus thaleichthys*, wakasagi *H. nipponensis*, and Mississippi silversides *Menidia audens*) co-occur with delta smelt and have a nearly identical body size and shape, which complicates species differentiation. Because of these challenges, sampling with trawled nets has been the only feasible method of studying delta smelt ecology in the wild.

**Figure 2:**
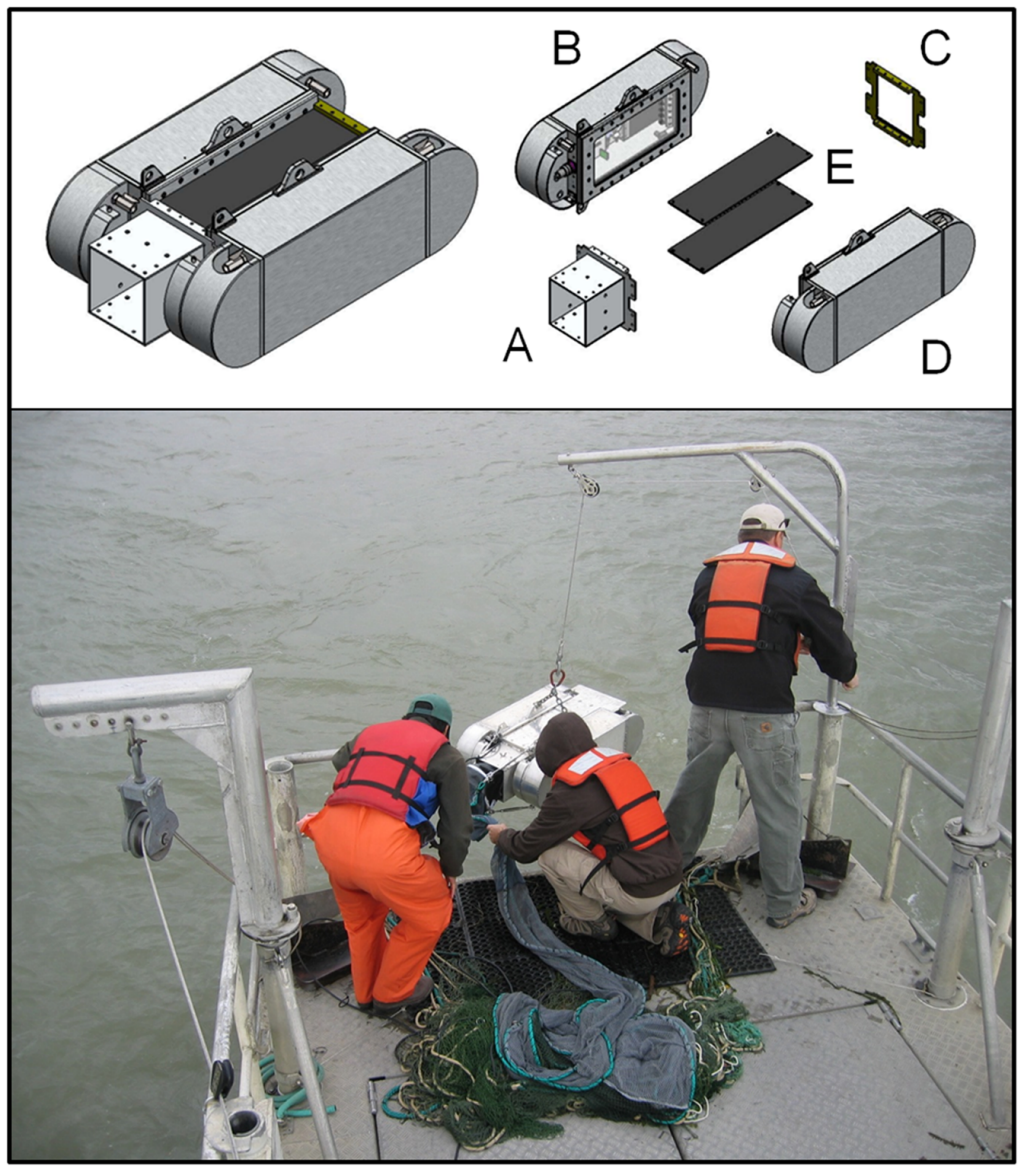
The SmeltCam. Upper panel is a diagram of the SmeltCam showing (A) net cowling and bow frame, (B) sealed electronics compartment, (C) stern frame, (D) ballast hull and (E) top and bottom vision tube covers. Bottom panel is a photograph of the SmeltCam being deployed from a research vessel.

Due to their extremely low abundance and delicate nature, continued study and monitoring of delta smelt poses a considerable challenge for scientists and managers. To overcome such challenges, resource agencies have invested in developing the SmeltCam, an underwater video camera codend for trawled nets (Fig. 2). Here, we describe the SmeltCam and its first application to advancing the understanding of delta smelt ecology. The purpose of our study was to better understand the fine-scale distribution of delta smelt. Our study question was: does the vertical and horizontal distribution of delta smelt vary by tide stage? The answer to this question is relevant for many reasons, including the opportunity to generate more precise population estimates of delta smelt [24]. Long-term fish monitoring in the upper San Francisco Estuary is not designed to generate actual population estimates of fishes. Rather, it generates dimensionless interannual indices of relative abundance. Sample design-based population estimates for delta smelt have been generated from the available monitoring data but are necessarily subject to its biases and limitations [24]. Three of the key issues are that sampling takes place (1) generally at center channel, (2) irrespective of tide stage, and (3) with the net towed obliquely through the water column. These issues present problems in extrapolating the trawl catches volumetrically to generate sample design-based population estimates because it is not known how delta smelt are distributed across tides vertically or horizontally in the water column. Another immediate application of our study is that the knowledge obtained on fine-scale habitat use can be used to inform behavior models examining the distribution and movements of delta smelt.

## Methods

### SmeltCam

The SmeltCam functions as an open-ended codend that automatically collects information on the number and species of fishes that pass freely through a trawled net. Key components of the SmeltCam include a bridle system that connects to a trawled net, a water-tight electrical housing and a ballast hull (Fig. 2).

The SmeltCam body is a combination of welded sheet aluminum and machined plate aluminum. The overall dimensions of the unit are 93 cm (length)×56 cm (width)×38 cm (height). In the configuration used in our study, a 4-point bridle system with turnbuckles and shackles was used to attach the SmeltCam to the four load-bearing lines of the trawled net. The unit weighs 48.5 kg dry and valves are used to adjust and maintain water levels in portions of the hollow ballast hull in order to achieve neutral buoyancy. The addition of ballast water adds considerably to the unit’s weight and necessitated a davit to lift it from and to the deck during and after deployment. The interior chamber of the SmeltCam where fish and other objects pass is 76.2 cm (length)×18.9 cm (width)×18.9 cm (height).

The starboard side of the unit contains the sealed electrical housing chamber. Within the electrical housing chamber are components and sensors that control and/or monitor positioning, physical conditions, lighting, and video functions. A global positioning system (GPS; uBlox-6 chipset, U-blox America Inc, San Jose California, USA) records position coordinates with an approximate 3 meter level of accuracy. A pressure gauge within the unit records depth with a vertical resolution of approximately 8 cm. Accelerometers measure the tilt, roll, and pitch of the device in the water. Sensors measure the interior computer and air temperature. Relative humidity is also measured to detect failure of the chamber seal and exposure of electronics to water.

The internal wall of the sealed electrical housing chamber is a 43 cm (length) × 25 cm (height) acrylic window that serves as the viewing area for the video system. The viewing area is essentially the interior chamber of the unit and thus has the same dimensions. Custom 8,000 lumen white LED is used to cast a wide swath of light to blanket the entire viewing window with even, wide angle of incidence lighting that reduces backscatter and specular reflection off of passing fish and other objects. A grayscale camera (Genie HM1400; Teledyne Dalsa, Billerica, Massachusetts, USA) mounted on the inside of the sealed electrical housing chamber captures 1600 ×1200 megapixel resolution images at a rate of sixty frames per second. The system is powered by 120 V AC 60 Hz, supplied by a 3000 W Honda portable generator, however on-board ship power is also suitable. Internal power supplies converted the 120 V AC power to 24 V DC and 12 V DC power. Power and communications to components in the sealed electrical housing chamber are provided by a simple, flexible, 3-conductor 14AWG waterproof cable, 180 m in length. Communications are sent up and down the same cable via powerline communications, in which TCP/IP packets are encoded on top of the 120 V AC power. The communications link over the 180 m distance is approximately 20 mbits/second.

Custom software has been developed to operate the system and to record data, which is operated with a standard laptop computer. A series of algorithms control object detection, tracking and identification. Object detection and tracking algorithms utilize gradient contour methods from raw image information obtained from the camera. Species identification is accomplished through algorithms in a support vector machine (SVM). The system uses several feature vectors to uniquely describe each species. The feature list includes object size, size and shape-independent list of shape moments (Hu moments), aspect ratio, defect from pure ellipse, RMS error (or deviation from) normalized species image, and radial local pattern. The SVM takes in all of the features and generates species identifications with an associated level of confidence for each object passing through the field of vision.

Training the SVM algorithm to identify fish species is an ongoing exercise and involves using positively identified images and metadata. Two separate cross-validation efforts were completed prior to conducting to this field study. The initial effort involved a training sample of human-identified images of delta smelt, threadfin shad *Dorosoma petenense* and American shad *Alosa sapidissima* acquired from the field in September 2011 to classify 306 images acquired in October 2011. Classification success was 88% for delta smelt, 56% for American shad and 37% for threadfin shad. We also conducted a k-fold cross validation using all fifty of the field-collected images of delta smelt obtained over the lifetime of the SmeltCam. The library of images was divided into 10 subsets (k = 10) where for each subset 10% of the images was used as a training set to identify the remaining 90% of the images. The average success rate over the 10 subsets was 91%, meaning that the algorithm could positively indentify 91% of the images that a human could positively identify. Online training is planned for the next phase of development, in which every positively identified object that passes through the device helps to improve the SVM algorithm. Algorithms are available upon request (inquiries should be sent to darren.odom@sureworksllc.com). While the algorithm works relatively well, it is continually being improved. Hence for this study we reviewed each image obtained during sampling and provided a relatively subjective human-assigned level of confidence for each species identification.

All system components and live video from the camera can be monitored in real time on board the research vessel and simultaneously written to file. Ultimately, each fish passing through the field of vision is given a species identification with an associated level of confidence, and all other sensor data is also recorded including date, time, GPS coordinates and depth. All images are also recorded and, as in the case of our study, can be reviewed for accuracy.

### Delta Smelt Ecology

The delta smelt was listed as a threatened species under both the California and Federal Endangered Species Acts in 1993. The listing status was changed to endangered by California in 2009. In 2010, a Federal status review determined endangered status was warranted but precluded by other higher priority listing actions.

Delta smelt abundance has been variable but has exhibited a substantial long-term decline (Fig. 1) [16], [24], [25]. The delta smelt is one of four fish species in the estuary which have exhibited further step-declines in about 2002 and have remained near all time record lows for the last decade, defining an era in the ecosystem known as the pelagic organism decline [18]. Long-term trends in abundance of delta smelt and other fishes are generated from data collected by the California Department of Fish and Wildlife’s (CDFW) Fall Midwater Trawl Survey (FMT), which has been conducted each year since 1967, with the exception of 1974 and 1979.

The delta smelt is an opportunistic carnivore that feeds primarily on planktonic copepods, cladocerans, mysids, and amphipods. It is primarily an annual species with very few individuals living and spawning a second year. Spawning takes place during spring in freshwater tidal habitats [17], [26]. Young delta smelt move downstream with the tides until they reach favorable rearing habitats in the low salinity zone (∼1–6) of the estuary [27], although some apparently remain in upstream reaches year-round [26]. During the summer and fall, juvenile delta smelt live primarily in the upper San Francisco Estuary associated with the low salinity zone [17], [23]. In winter delta smelt migrate upstream to freshwater habitats where spawning occurs during spring.

### Study Area and Design

**Figure 3:**
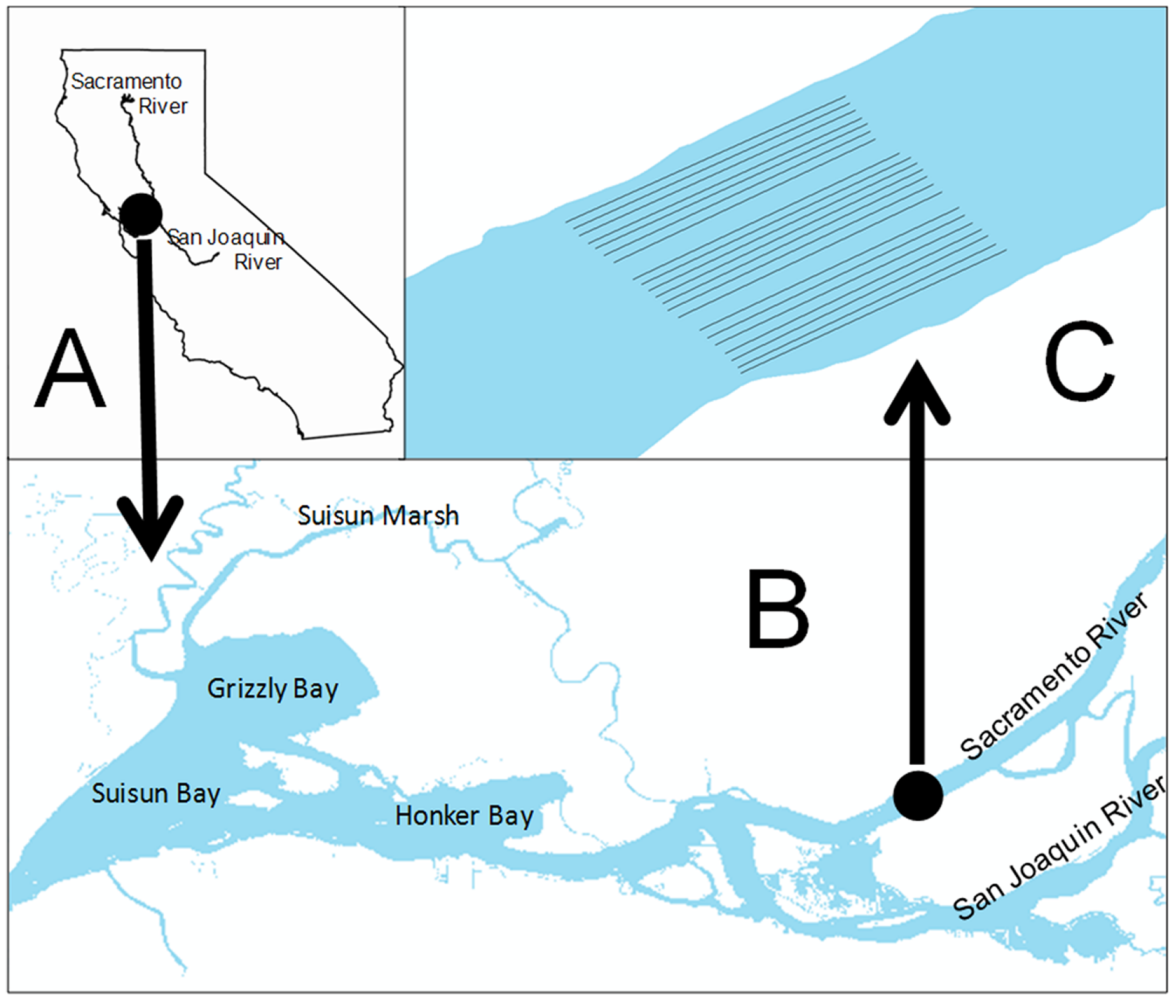
Map of the study area showing the (A) location of the upper San Francisco Estuary in California, (B) location of the study site in the upper estuary, and (C) orientation of trawling lane transects in the tidal Sacramento River channel.

Our study focused on the fine scale distribution of juvenile delta smelt in the upper estuary during fall. Field sampling for delta smelt was conducted under a permit granted to the Interagency Ecological Program by the U.S. Fish and Wildlife Service. We conducted our study November 27–30, 2012 in the Sacramento River adjacent to Sherman Island, approximately between routine FMT stations 704 and 705 (Fig. 3). We chose this region because: (1) long-term FMT monitoring data indicate delta smelt remain relatively abundant in this area due to suitable habitat conditions [23], (2) two other studies, one examining movements of delta smelt (J. Burau, U.S. Geological Survey, Sacramento, California, personal communication) and another examining sampling efficiency of several different trawled nets (R. Baxter, unpublished data), effectively sampled delta smelt in this area in the two months preceding our study, September-October 2012, and (3) routine FMT sampling in the three months preceding our study also detected delta smelt in the area.

**Figure 4:**
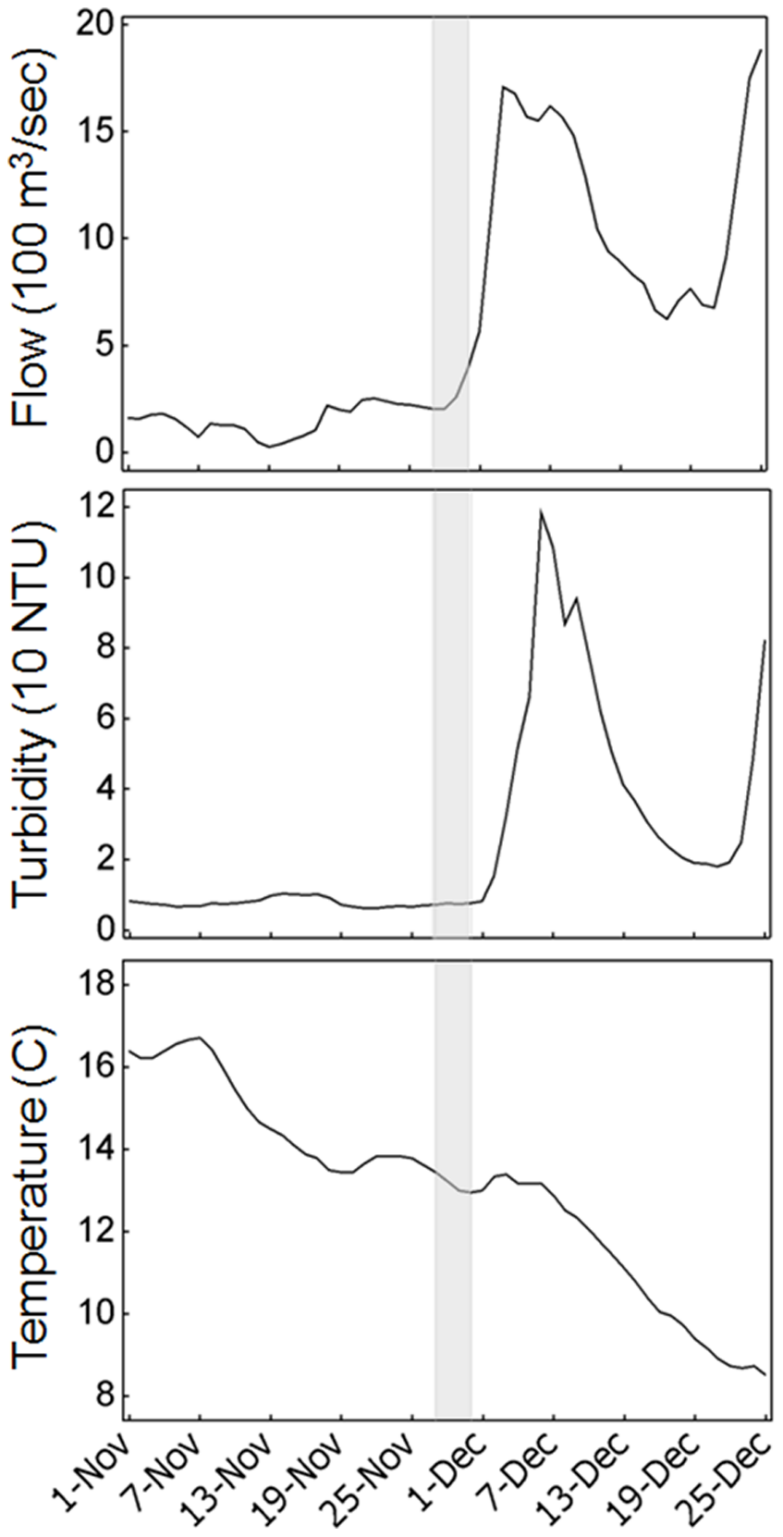
Seasonal time series of flow, turbidity and water temperature with the study period shaded in gray.

Physical conditions during our study were typical for the region during the fall but dramatically changed shortly thereafter (Fig. 4). During the study a storm moved across California and produced a moderate amount of intermittent rain and south winds in the immediate study area. The storm persisted after our study and within two weeks produced what is colloquially termed “first flush” conditions, which refers to the initial onset of substantially elevated river flows and turbidity entering the estuary (Fig. 4; data obtained from the California Data Exchange Center http://cdec.water.ca.gov/). These conditions are associated with the upstream migration of delta smelt to areas where spawning ultimately occurs during spring [26], [28]. Our study, therefore, observed fine-scale delta smelt distribution patterns during typical fall conditions prior to the “first flush” and the upstream migration of delta smelt.

We conducted our study using the same equipment (e.g., research vessel, net, and crew) as is normally used for the FMT. The only exception was that the SmeltCam was affixed to a slightly modified codend of the net rather than the codend being tied closed. Descriptions of the standard FMT protocol and sampling sites are readily available [23], [29]. The net itself is 17.6 m long with a square mouth opening of 3.66 m in width and height. It has nine tapered panels of stretch mesh from 14.7 cm near the mouth to 1.3 cm in the codend. To generate the data used to calculate the interannual indices of relative abundance, the FMT collects samples via a 12-minute oblique trawl conducted at 100 sites distributed across the tidal freshwater to mesohaline regions of the estuary each month from September to December. To attach the SmeltCam, the codend of the net was modified by slightly adjusting the lengths of the last two mesh panels and attaching an additional panel of 0.64 cm knotless mesh measuring 74.9 cm in length sewn to a 7.6 cm-diameter vinyl collar attached to the SmeltCam housing. These modifications increased the total length of the net from 17.6 m to 17.8 m.

As mentioned above, long-term FMT data and recent research efforts helped guide our study design. The aforementioned study examining delta smelt movements influenced our experimental design with its observation that delta smelt were collected during flood tides but rarely during ebb tides in surface samples taken with a Kodiak Trawl in both 2010 and 2012. Expanding upon that observation and to more closely examine the position of delta smelt in the water column, we set up a factorial study design with three factors and two levels for each factor, thus 2^3^ = 8 possible treatments. The three factors and their corresponding levels were: (1) horizontal position in the water column (H: center of the channel versus side of the channel), (2) vertical position in the water column (V: upper half versus lower half) and (3) tidal phase (T: flood versus ebb). The response variable, fish counts, was defined as the number of delta smelt collected in a 10-minute trawl. Since counts are functions of density and volume sampled, and our interest was in how density varied by treatment combination, the volume of water filtered by the trawl was estimated using a mechanical flowmeter (model 2030R, General Oceanics, Inc.) deployed off the side of the research vessel during each trawl.

Available time and resources facilitated the day-time collection of fifty-six samples over a four day period, thereby allowing seven replicates per treatment combination. To determine the adequacy of these sample sizes, we estimated, given seven replicates and a standard deviation for fish counts of 2.1 (based on thirty-five FMT samples taken during the previously mentioned net efficiency study), that there was a 95% probability of rejecting a test of the null hypothesis of no factorial effects, i.e., the expected fish counts are the same for all treatment combinations, when at least one of the combinations had an expected catch that was three fish above (or below) that for other combinations. Further, pairwise differences in fish counts as small as three would be detected with 79% probability and as large as four would be detected with 95% probability.

We used a combination of GIS (geographic information system) and GPS to select and occupy sampling locations in the Sacramento River channel in order to achieve our study objective. GIS (ArcGIS 9.3.1, ESRI, Redlands, California, USA) was used to generate a total of twenty-one possible equidistant trawling lanes oriented longitudinally in the channel; the first and last seven lanes represented the sides of the channel while the middle seven lanes represented the center of the channel (Fig. 3). Sides of the channel were treated as a single unit and the specific side sampled was determined randomly. The number of lanes and their spacing were generated so that trawling in one lane would have no effect on adjacent lanes. The lanes were loaded into a GPS unit and tracked by the research vessel during sampling. Water depth averaged 10.5 m during ebb tides and 10.1 m during flood tides. Sampling depth (upper half versus lower half of the water column) was achieved by maintaining the net either above or below mid-depth during a trawl, targeting ¼ or ¾ depth (∼2.6 or 7.9 m), respectively. Water depth was determined by a boat-mounted sonar unit. Net depth was adjusted with the length of cable between the net and the boat and determined by the SmeltCam’s depth sensor, which was monitored in real-time during trawls.

A ten-minute sample was recorded for each trawl once the net and SmeltCam were positioned at the appropriate depth. The water filtered by the net during the time it took to go from the surface to the appropriate depth at deployment and then back again at retrieval was not considered part of the sample and was not recorded. For consistency, trawls were done so that the net was towed longitudinally in the channel against the current (i.e., upstream during ebb tides and downstream during flood tides). The order in which lanes and depths were sampled was randomly generated. Sampling necessarily had to follow the order of the tides. We examined forecasted tidal velocities generated from the CALSIM Hydrologic Model [30] to appropriately arrange sample collection around the tides. Sampling took place only during daylight hours, consistent with FMT protocol.

We measured water temperature (°C), salinity, turbidity (NTU), Chl *a* concentration (µg/L), pH and dissolved oxygen concentration (mg/L) immediately preceding and following each trawl in both the upper and lower half of the water column. Spot measurements were taken with a handheld YSI multiparameter sonde rigged with a communication cable long enough to reach the appropriate depth (YSI Inc, Yellow Springs, Ohio).

To statistically evaluate the effects of the three main factors (tide, horizontal and vertical position in the water column) on delta smelt density and the water quality variables, we fit several models commonly used for count data. In particular, we fit log linear Poisson models, models allowing for overdispersion (the quasi-Poisson and negative binomial), and models allowing for excess zeros (the zero inflated negative binomial), where overdispersion and excess zeros are with reference to the Poisson distribution [31]. Model fitting was done using the statistical computing environment R, version 2.15.1 [32], along with the R package ‘pscl’ [33], [34]. To make between model comparisons, we calculated AIC values, AIC = 2*k –2*log(Likelihood), where k = the number of parameters. AIC simultaneously quantifies goodness of fit, as defined by the likelihood of the data, and model complexity (as measured by k), and models with the smallest AIC values are considered preferable [35]. For each model, P-values for factors and factor combination were also calculated to assess the significance of particular factors.

## Results

We collected 52 samples during the four days of field study; mechanical problems with the research vessel prohibited us from completing our intended number of replicates for each treatment (Table 1). In total we collected 30 samples during flood tides and 22 samples during ebb tides. Due to variations in tidal velocities the volume of water sampled per 10-minute trawl varied from 4,388 m^3^ to 8,057 m^3^, but on average was comparable across the eight treatments (Table 1).

**Table 1:** Average±one standard deviation of the water volume (m^3^) sampled across the eight study treatments.

		Flood tide	Ebb tide	
Top half of the water column				
	Middle channel	5,543±312 (7)	5,860±370 (6)	
	Side channel	5,781±780 (8)	5,587±170 (5)	
Bottom half of the water column				
	Middle channel	5,616+154 (7)	6,929±683 (6)	
	Side channel	5,656±803 (8)	6,741±1,150 (5)	

Sample sizes are provided in the parentheses.

**Figure 5:**
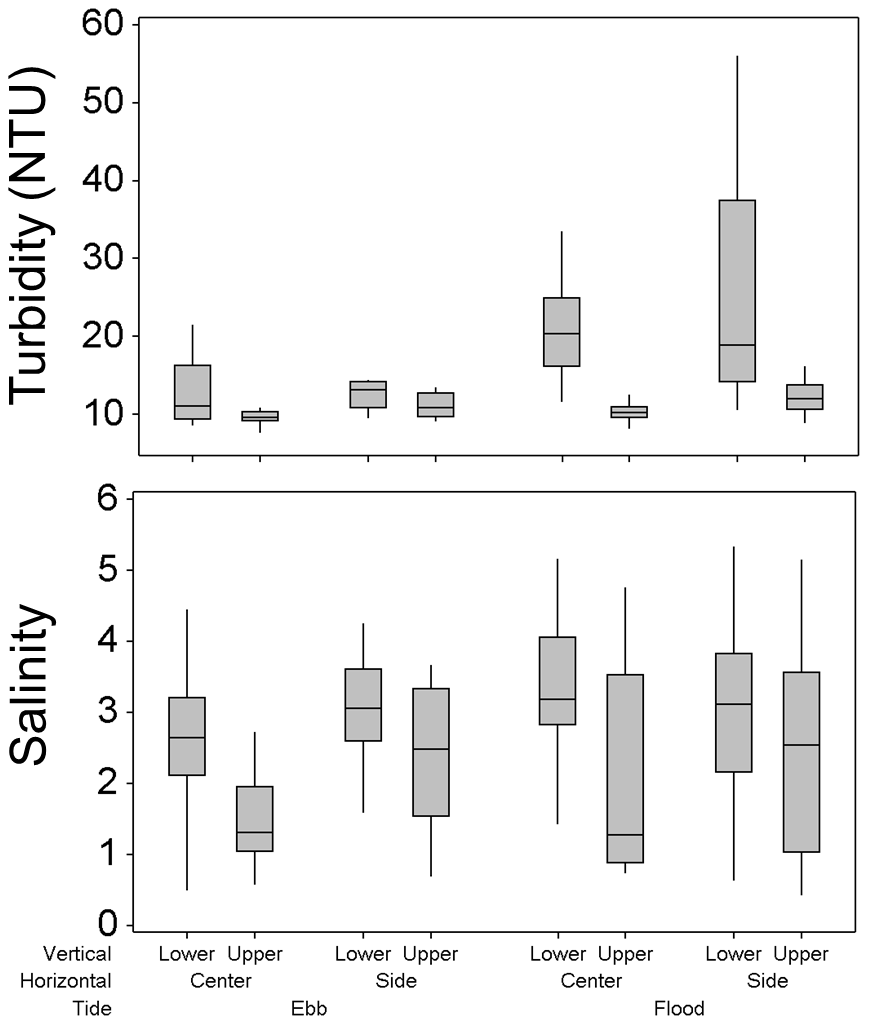
Box plots of turbidity and salinity by tide and position in the water column.

Water temperature averaged 13.8°C and varied by less than 1°C during the entire study (minimum = 13.5°C, maximum = 14.2°C). Dissolved oxygen concentration ranged from 8.4 mg/L to 8.9 mg/L, pH ranged from 7.4 to 7.8 and chlorophyll *a* concentration ranged from 1.2 µg/L to 3.5 µg/L. Given their low variability, these water quality variables were not included in subsequent analyses. Salinity averaged 2.6 and ranged from 0.4 to 5.3. As determined by a standard generalized linear model, salinity differed significantly (P<0.05) with horizontal position across the channel and vertical position in the water column. Salinity averaged about one unit higher in the center of the channel versus the side of the channel, and also on the bottom half of the water column versus the upper half of the water column (Fig. 5). Salinity did not differ across tides because we sampled the full tidal cycle. Consequently, salinity values expectedly overlapped during ebb and flood tides. Turbidity averaged 15.7 NTU and ranged from 7.6 NTU to 80 NTU. Turbidity exhibited statistically significant differences (P<0.05) among all combinations of factors and their interactions except for the tide:horizontal position, horizontal position:vertical position and tide:horizontal position:vertical position interactions. The most striking pattern with turbidity was that it was higher in the lower half of the water column, and substantially higher during flood tides (Fig. 5). Both salinity and turbidity were lowest during ebb tides in the center of the channel in the upper half of the water column.

**Figure 6:**
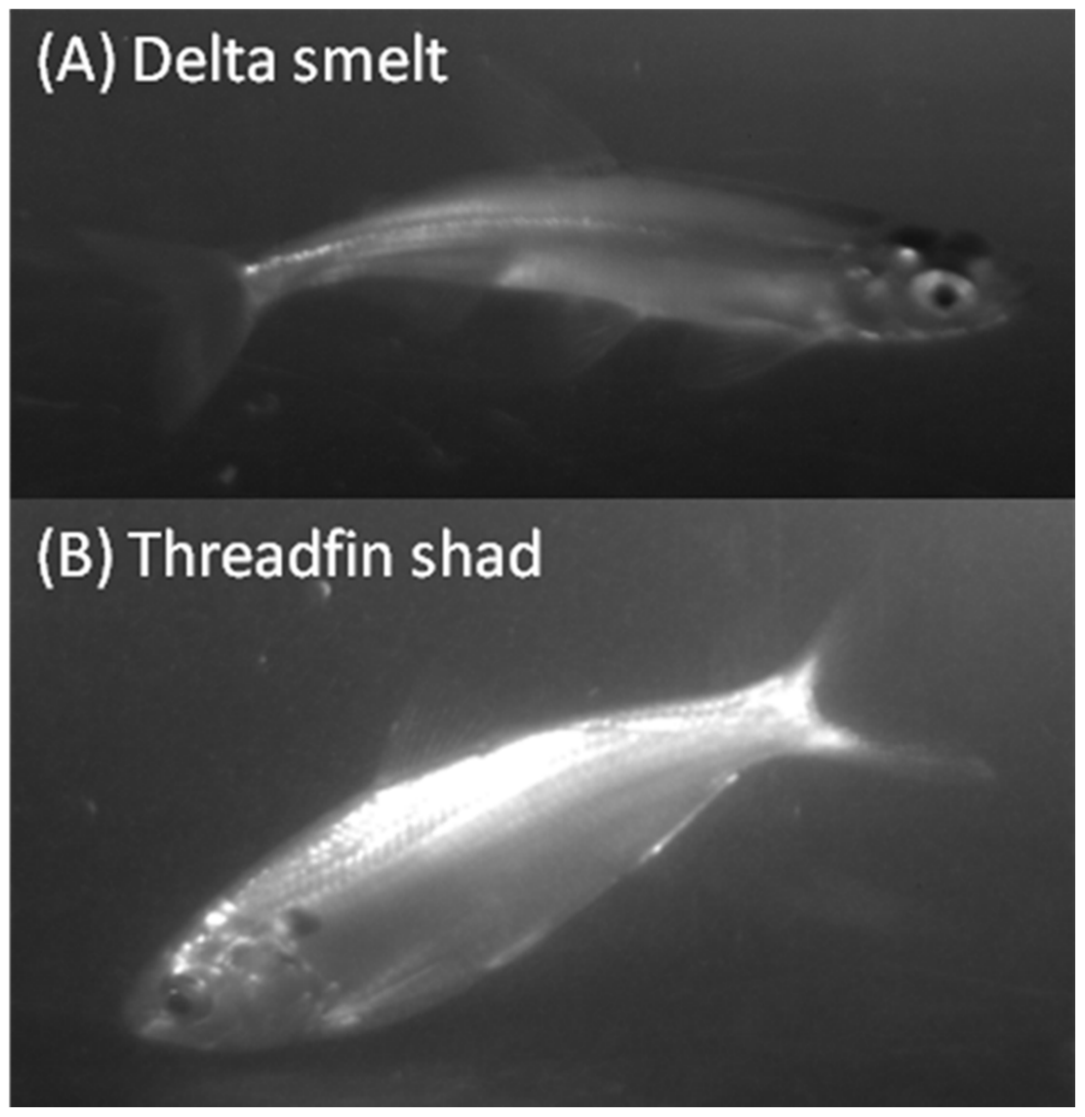
Examples of raw images of (A) delta smelt and (B) threadfin shad obtained by the SmeltCam during our field study. Note that in the delta smelt image all of the fins, including the adipose fin, are clearly visible and allow delta smelt to be differentiated from other species such as longfin smelt. Also note that the dorsal fin thread is visible across the caudal peduncle in the threadfin shad image.

**Figure 7:**
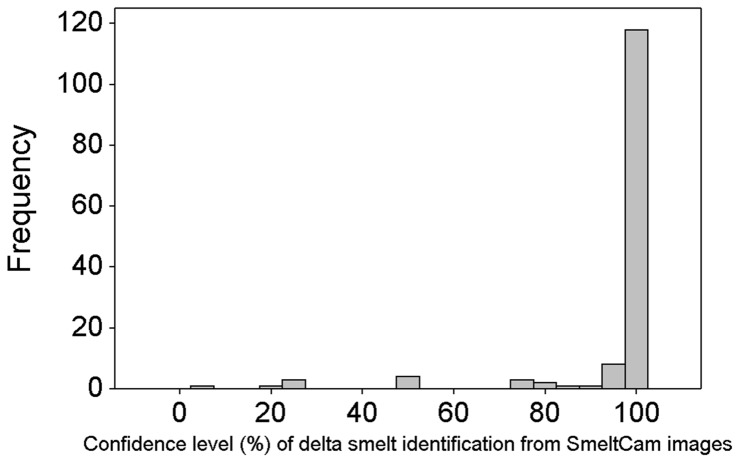
Frequency histogram of the confidence level (%) that delta smelt were correctly identified to species.

**Figure 8:**
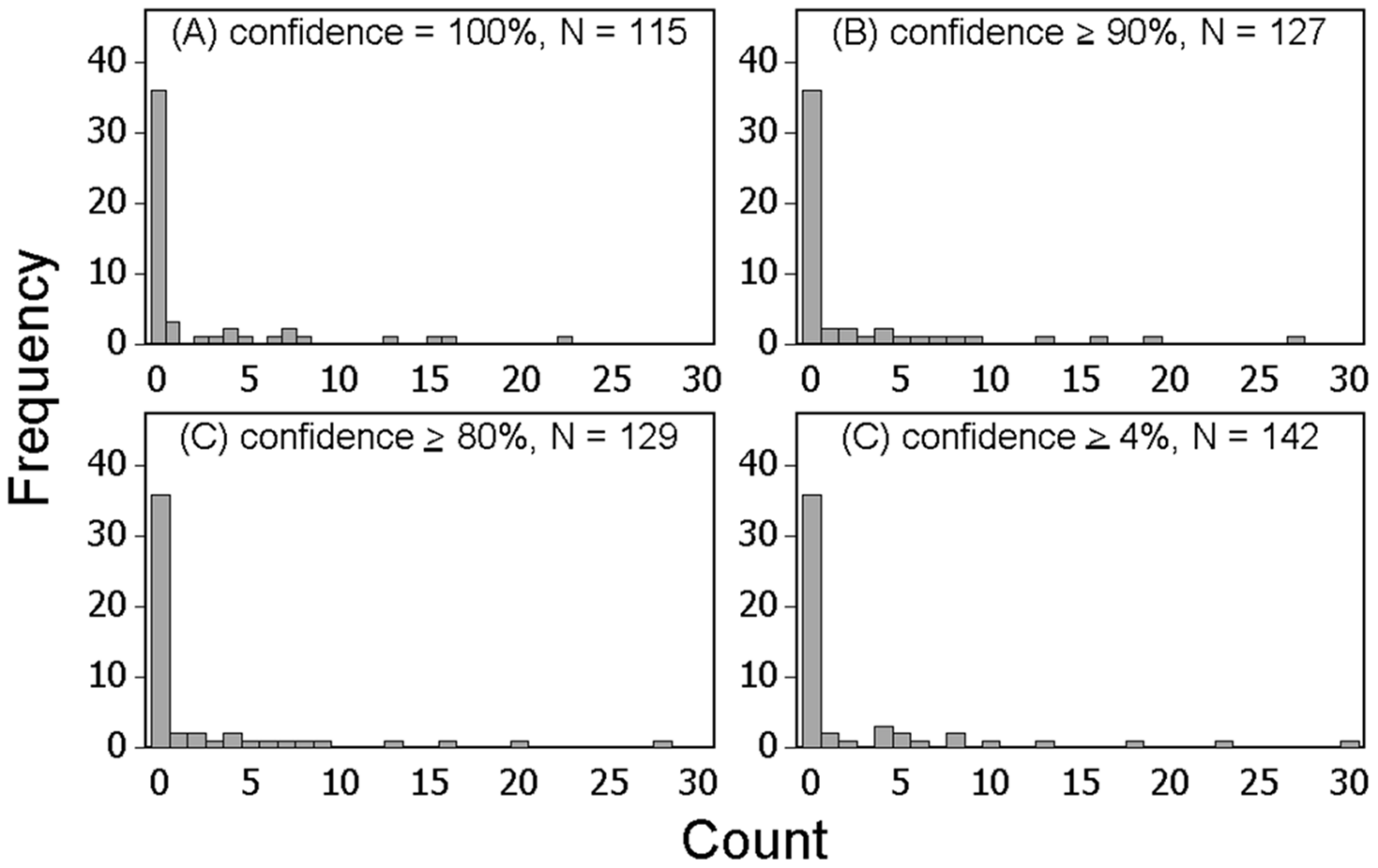
Frequency histograms of the count of delta smelt collected per sample for individuals that were correctly identified to species on SmeltCam images with (A) 100%, (B) ≥90%, (C) ≥80%, and (D) ≥4% confidence.

We collected 352 individual fish comprised of 6 different species during our study: green sturgeon *Acipenser medirostris* (1), starry flounder *Platichthys stellatus* (1), American shad (23), striped bass *Morone saxatilis* (43), threadfin shad (87), and delta smelt (197). Count data on all of the species except for delta smelt were insufficient for further analysis. Of the 197 delta smelt collected, 142 individuals swam through the SmeltCam (Fig. 6) while 55 were entangled in the mesh of the net. We measured the fork lengths of 29 of the 55 individuals that were found in the net; they ranged from 51 mm to 75 mm (average = 64.5 mm and standard deviation = 5.2 mm). Subsequent summaries and analyses focus on individual delta smelt observed by the SmeltCam. The level of confidence in the identifications (human-assigned) ranged from 4%–100%, with 100% comprising the majority of the values (Fig. 7). Because there was 100% confidence in most of the identifications, the sample distribution (count of individual delta smelt per sample) did not change when examined across varying levels of confidence in species identification (Fig. 8). Nonetheless, to err on the side of caution we focus our analyses hereafter on individual delta smelt that were identified to species with 100% confidence.

Delta smelt were observed in 16 of 52 samples, thus zero counts were observed in 69% of the samples. The number of delta smelt per sample ranged from 0 to 22 (average = 2.2, standard deviation = 4.7), while density ranged from 0 to 37/10,000 m^3^ (average = 3.8/10,000 m^3^, standard deviation = 8.3/10,000 m^3^). The average and standard deviation were more than two times higher than expected based on the aforementioned net efficiency study, which observed a mean of 0.9 delta smelt per sample and a standard deviation of 2.1.

**Figure 9:**
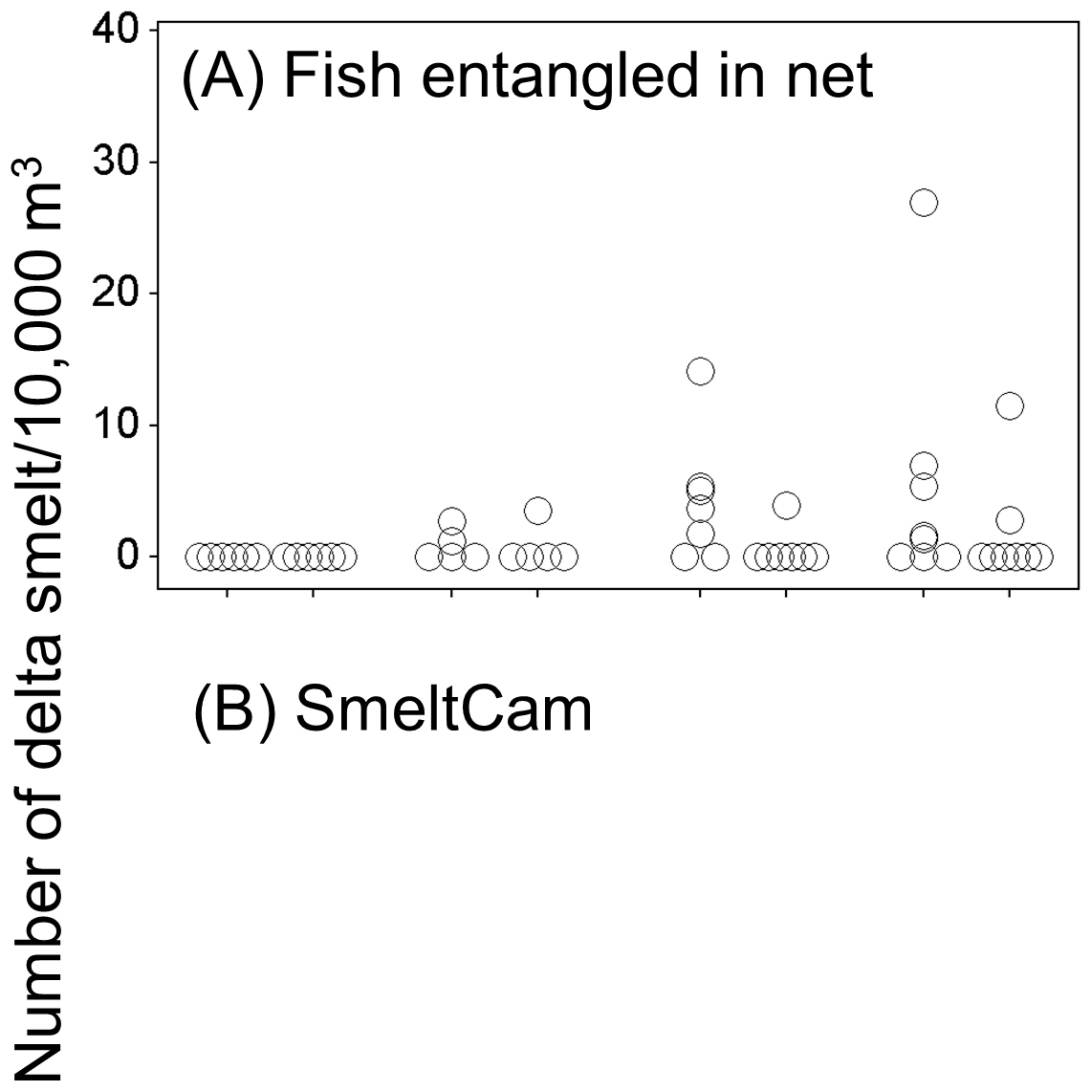
Individual values of delta smelt density (number/10,000 m^3^) by tide and position in the water column for fish that were (A) found entangled in the mesh of the net when it was retrieved and (B) observed by the SmeltCam.

The mean delta smelt density was 5.9/10,000 m^3^ for flood tides versus 0.8/10,000 m^3^ for ebb tides. For positive samples only (excluding the zero counts), the mean density was 14.9/10,000 m^3^ for flood tides versus 5.0/10,000 m^3^ for ebb tides. Delta smelt were observed throughout the water column on flood tides but only at the lower half and side of the channel on ebb tides (Fig. 9).

We fit the following families of models: Poisson (Po), quasi-Poisson (q-Po), negative binomial (NB), and zero-inflated negative binomial (ZINB). In each case the response variable was the observed number of delta smelt caught. We used a log link function to model the expected number of delta smelt caught. The expected number is the density times the volume sampled, and since the log of the volume sampled was handled as an offset, the factors were thus modeling expected delta smelt density. For each of the four model families, expected fish density was modeled using the following set of nested models (and the R syntax for the model formula):

1. Main effects only (H+V+T)
2. Main effects with 2-way interactions (H*V+H*T+V*T)
3. Main effects, 2-way and 3-way interactions (H*V*T)

The results are summarized in Table 2. The Poisson models found many more statistically significant factors and factor combinations than the other three families of models. However, this is due to relatively small, and likely too small, estimated variances (based on results for the other families of distributions and the fact that the Poisson models had the highest AIC values).

**Table 2:** Summary of model fitting results for the nested set of factors and four different families of distributions.

Factors	Po	Q-Po	NB	ZINB
H+V+T				
AIC	348	NA	164.5	162.8
Var	µ	7.6·µ	µ(1+µ·5.9)	0.15 µ (1+ µ·0.7)
Signif	H**,T**	T*	T**	T**
H:V+H:T+V:T				
AIC	313	NA	165.5	161.5
Var	µ	6.0·µ	µ(1+µ·4.8)	0.15 µ (1+ µ·0.4)
Signif	V**,T**,V:H**	T*	T**	T**
	V:T*,H:T*			
H*V*T				
AIC	314	NA	167.2	162.9
Var	µ	6.0·µ	µ(1+µ·4.7)	0.15 µ (1+ µ·0.4)
Signif	T**,H:T*	T^’^	–	T**

H+V+T is main effects only, H:V+H:T+V:T is main effects and 2-way interactions, and H*V*T is main effects, 2-way and 3-way interactions. Po = Poisson, Q-Po = Quasi-Poisson, NB = Negative Binomial, ZINB = Zero Inflated Negative Binomial. The Var entries refer to the variance function for the distribution families. The Signif entries denote the significant factor effects, with superscripts ‘,*, and ** denoting significance at the 0.10, 0.05, and 0.01 levels, respectively. The reported AIC value for the Q-Po case is the Quasi-AIC value.

The quasi-Poisson model provides estimates of the inflation of the variances relative to the Poisson, e.g., overdispersion multipliers of 6.0 to 7.6. AIC values are not calculated for quasi-Poisson models because there is no likelihood. Quasi-AIC values have been developed [35] but they are of use only for comparing different quasi-Poisson models, not for comparing differences between families of distributions. Restricting attention to just the quasi-Poisson combinations, with the enlarged variance only the main effect of tide was found statistically significant for all three factorial combinations.

Amongst the negative binomial models, the main effects model has the smallest AIC value. The AIC value for the two-way interaction model is quite similar and negligible based on a rule of thumb [35], i.e., a difference of 2 units or less is not important. Given two models with negligibly different AIC values, the simpler model is preferable. Like the quasi-Poisson, the negative binomial model also increases the variance relative to the Poisson. Ver Hoef and Boveng [36] address the question of choosing between quasi-Poisson and negative binomial models and note that the key distinction is the nature of the variance function. The negative binomial variance is µ(1+ µ/θ), the multipliers shown in Table 2 are 1/θ, in contrast to the quasi-Poisson µ·φ, where φ is the overdispersion parameter. Ver Hoef and Boveng [36] suggest plotting (y- µ)^2^ against µ to select between the two families. Such plots were produced and the relationships were quite similar for quasi-Poisson and negative binomial and failed to indicate a preference for one family over the other. We note that the estimated coefficients for the tide effect were quite similar for quasi-Poisson (1.8) and negative binomial (2.0), so choosing between the two families does not seem critical.

Based on AIC values, the zero inflated binomial models are the best of the three families for which AIC can be calculated, with the main effects model our preference (based on the above argument on comparing AIC values). Zero inflated models are mixture models of the following general form: π f(0)+(1- π) f(non-negative), where π is the probability that the data come from the degenerate distribution, f(0), where 0 is the only possible outcome and f(non-negative) is the probability distribution allowing 0′s and positive outcomes. The probability π was modeled according to a simple logistic model, log(π/(1- π) = ⌈, while the negative binomial distribution was used for f(non-negative) with the expected counts modeled as functions of the factor levels. For all three sets of factorial combinations, π, was 0.61, i.e., there was at least a 61% probability of failing to catch any delta smelt (the probability of no delta smelt also includes the case where the negative binomial model yields a zero). This value seems reasonable given the observed 69% of zeros in the catches. Like the quasi-Poisson and negative binomial models, the sole significant factor was the main effect of the tide, with a similar coefficient of 1.7 for the flood level effect.

## Discussion

Our results suggest that the distribution of juvenile delta smelt in the water column varied across tides. We found that delta smelt were relatively common and abundant throughout the water column during flood tides. However, during ebb tides delta smelt were less abundant, and were observed only in the lower half of the water column and sides of the channel. This pattern emerged from both the fish observed by the SmeltCam and those that were found entangled in the mesh of the net after it was retrieved. With regard to this specific pattern, there is no bias associated with not knowing the exact depth at which the fish entangled in the mesh of the net were captured because none were captured during ebb tides in the center of the channel (Fig. 9). Interestingly, variability in salinity and turbidity exhibited the same general pattern as did delta smelt, and may be the proximal reason for the distributions observed. The performance of the SmeltCam degrades when turbidity exceeds approximately 80 NTU. Turbidity reached 80 NTU in one of fifty-two samples we collected; a flood tide treatment from the lower half of the water column and side of the channel. Seven delta smelt were observed in this sample. It is possible that more delta smelt were actually present but were missed by the SmeltCam because of the elevated turbidity. If this were true, it would be consistent with the overall pattern of delta smelt distribution and would not have changed the results. Salinity and turbidity are both important components of delta smelt physical habitat [22], [23]. We hypothesize that delta smelt, by simply remaining within preferred turbidity and salinity conditions across tides, could have produced much of the pattern observed. However, it appeared that delta smelt manipulate their position in the water column either through keying in on these water quality conditions or the physics underlying them.

Our results were consistent with other studies in 2010 and 2012 that found delta smelt to be abundant on flood tides but not on ebb tides (J. Burau, U.S. Geological Survey, Sacramento, California, unpublished data), although the studies were conducted under very different net flow conditions. The general consistency in results across studies in multiple years and the observation that physical habitat is a likely underlying mechanism, together provide strong evidence that delta smelt were not randomly distributed in the water column across tides. However, the extent to which the pattern observed at this location holds true at night or at other locations is uncertain. Although we did not sample at night, the delta smelt movements study did and found no difference in catch patterns compared to the day.

There is sufficient evidence to suggest that the patterns of delta smelt distribution observed in this particular location are not applicable across the entire system. Our unpublished analyses (separate independent analyses by FF, KBN and DS) of the FMT data set demonstrate a high degree of variability in delta smelt catches across tides among sampling sites. It therefore seems likely that localized physical as well as biological components of habitat influence delta smelt distribution across the system. The likelihood that delta smelt distribution in the water column across tides varies spatially in the system has important implications for the original motivation for our study. We generated information relevant to informing methods that could be developed to extrapolate survey data to generate more precise population estimates of delta smelt. However, it is clear that additional study is needed to characterize variability at other locations in order to successfully revise present methods of estimating delta smelt population size. Areas of particular interest are the broad expansive shallow water shoals located in Suisun, Grizzly and Honker bays (Fig. 3).

There is also sufficient data to suggest that the patterns of distribution we observed do not hold true for other life stages of delta smelt. A previous study of delta smelt larvae found no effect of tide on vertical distribution [37]. Similar to above, our unpublished analyses (separate independent analyses by KBN and DS) of post-larval and juvenile delta smelt long term monitoring data sets (CDFW’s 20 mm and Summer Townet Surveys) demonstrate a high degree of variability in delta smelt catches across tides among sampling sites. Interestingly, previous studies demonstrated that the larvae of several native and exotic fishes (other than delta smelt) in San Francisco Estuary appeared to be behaviorally flexible in maintaining vertical position under different environmental conditions to maximize retention [38]. Together, these observations suggest that fish distribution in the water column varies according to localized habitat conditions.

Tidal movements, migrations and transport are well documented in systems worldwide and are usually associated with exploiting favorable habitats [39], [40]. Invertebrates such as penaeid shrimp are well known to selectively move or migrate with tides [41] as are fish. For example, flounder larvae *P. flesus* entered the water column on flood tides to move upstream into the Elbe River Estuary, Germany [42]. Similarly, plaice larvae *Pleuronectes platessa* accomplish passive but selective horizontal transport by entering the water column during flood tides and remaining on the bottom during ebb tides [43]. Studies in a tropical tidal mangrove have also shown that fish were distributed on the bottom during ebb tides and entered the water column during flood tides to exploit intertidal habitats [44]. For delta smelt, it appears that individuals manipulate their position in the water column to facilitate either movement or retention at different life stages. As alluded to above, we believe that the patterns we observed for juvenile delta smelt facilitate retention in favorable habitats. However, upstream migration of adults and downstream migration of larvae is undoubtedly facilitated by tidal transport and net flows. As mentioned, a previous study found no effect of tide on the vertical distribution of delta smelt larvae [37], suggesting they may be passively transported downstream by net flows until reaching favorable habitats near the low salinity zone where they effectively maintain position [27], potentially by manipulating their position in the water column. As a case in point, anadromous rainbow smelt *Osmerus mordax* larvae are known to vertically migrate to maintain position in regions of high prey density [45], [46]. The ability of young fishes to change their vertical distribution ontogenetically [47], [48] or in response to varying net flow conditions [38] appears to be a common strategy for retention in favorable habitats.

There are several advantages and disadvantages of the SmeltCam versus similar camera systems that have been used previously to photograph objects passing through the codend of nets. Silhouette photography or video recording devices have been developed for examining plankton distributions at various scales [3], [6], [9], [49], [50]. Perhaps the biggest limitation of the present generation SmeltCam is that it has been designed for a particular size range of fish. Modifications to the system could be made for sampling large-bodied fishes with larger nets or for sampling smaller organisms such as fish larvae or other planktonic organisms. An advantage of the SmeltCam is the incorporation of new technology enabling rapid digital photography, automatic object recognition, automatic data collection, and real time observation. We are actively working on improving the system in several ways including redesigning the frame and hull to make the unit lighter and easier to handle by a single individual, enhancing software to improve and expand automatic image recognition and adding size measurements, incorporating tools to record water quality parameters in real time during sampling, and matching the unit with trawled nets with dimensions that will decrease the entanglement of fishes and improve survival.

One potential concern associated with the SmeltCam is the possibility of predation occurring inside the unit given its dimensions and light emission, both of which could potentially attract and congregate predators such as striped bass. Although we did not conduct an exhaustive investigation on the topic, we found no evidence of predation attributed to the SmeltCam during our study. Immediately upon collection we sacrificed and examined the stomach contents of 5 striped bass (that were either entangled in the net or couldn’t fit through the opening of the SmeltCam) that were large enough to consume fish. None of the stomachs contained any fish remains; one stomach contained one isopod and the other four stomachs were empty.

Our study demonstrates how new technology can be developed to address key questions and uncertainties in conservation biology, and that imperiled species can be studied with relatively little harm. During our study we observed a total of 197 individual delta smelt. Of this total, 142 individuals passed through the SmeltCam alive while 55 died as a result of getting entangled in the mesh of the net. Thus, with the application of the SmeltCam in this study we increased the survival of individual delta smelt by 72% compared to using a traditional codend where all of the fish would have likely died due to handling stress. Survival is likely to increase in future studies as the SmeltCam is matched with nets with dimensions that will decrease entanglement. The SmeltCam can be affixed to virtually any type of trawled net or other fish congregating device facilitating a broad array of potential applications. The development and application of new technology such as the SmeltCam provides many new opportunities to studying imperiled species such as delta smelt and can be readily applied to other species and systems.

## References

[1] Thompson W (Editor) (2004) Sampling rare or elusive species: concepts, designs, and techniques for estimating population parameters. Island Press.

[2] MacKenzie DI, Nichols JD, Sutton N, Kawanishi K, Bailey LL (2005) Improving inferences in population studies of rare species that are detected imperfectly. Ecology 86: 1101–1113.

[3] Lenz J, Schnack D, Petersen D, Kreikemeier J, Hermann B, et al (1995) The Ichthyoplankton Recorder: a video recording system for in situ studies of small-scale plankton distribution patterns. ICES Journal of Marine Science: Journal du Conseil 52: 409–417.

[4] Swann DE, Cutler TL (1999) Using Remote Photography in Wildlife Ecology: A Review. Wildlife Society Bulletin 27: 571–581.

[5] Swann DE, Hass CC, Dalton DC Wolf SA (2004) Infrared-Triggered Cameras for Detecting Wildlife: An Evaluation and Review Wildlife Society Bulletin. 32: 357–365.

[6] Culverhouse PF, Williams R, Benfield M, Flood PR, Sell AF, et al (2006) Automatic image analysis of plankton: future perspectives. Marine Ecology Progress Series 312: 297–309.

[7] Watson DL, Harvey ES, Anderson MJ, Kendrick GA (2005) A comparison of temperate reef fish assemblages recorded by three underwater stereo-video techniques. Marine Biology 148: 415–425.

[8] Willis TJ, Babcock RC (2000) A baited underwater video system for the determination of relative density of carnivorous reef fish. Marine and Freshwater Research 51: 755–763.

[9] Olney JE, Houde ED (1993) Evaluation of in situ silhouette photography in investigations of estuarine zooplankton and ichythyoplankton. Bulletin of Marine Sciences 52: 845–872.

[10] Morrison M, Carbines G (2006) Estimating the abundance and size structure of an estuarine population of the sparid *Pagrus auratus*, using a towed camera during nocturnal periods of inactivity, and comparisons with conventional sampling techniques. Fisheries Research 82: 150–161.

[11] Becker A, Cowley PD, Whitfield SK (2010) Use of remote underwater video to record littoral habitat use by fish within a temporarily closed South African estuary. Journal of Experimental Marine Biology and Ecology 391: 161–168.

[12] Frezza TL, Carl LM, Reid SM (2003) Evaluation of a portable underwater video camera to study fish communities in two Lake Ontario tributaries. Journal of Freshwater Ecology 18: 269–276.

[13] Daum DW (2005) Monitoring fish wheel catch using event-triggered video technology. North American Journal of Fisheries Management 25: 322–328.

[14] Jordan F, Jelks HL, Bortone SA, Dorazio RM (2008) Comparison of visual survey and seining methods for estimating abundance of an endangered, benthic stream fish. Environmental Biology of Fishes 81: 313–319.

[15] Ellender BR, Becker A, Weyl OLF, Swartz ER (2012) Underwater video analysis as a non-destructive alternative to electrofishing for sampling imperiled headwater stream fishes. Aquatic Conservation: Marine and Freshwater Ecosystems 22: 58–65.

[16] Moyle PB, Herbold B, Stevens D, Miller LW (1992) Life history and status of delta smelt in the Sacramento-San Joaquin Estuary, California. Transactions of the American Fisheries Society 121: 67–77.

[17] Bennett WA (2005) Critical assessment of the delta smelt population in the San Francisco Estuary, California. San Francisco Estuary and Watershed Science. Online serial: http://repositories.cdlib.org/jmie/sfews/vol3/iss2/art1.

[18] Sommer T, Armor C, Baxter R, Breuer R, Brown L, et al (2007) The collapse of pelagic fishes in the upper San Francisco Estuary. Fisheries 32: 270–277.

[19] Mac Nally, R ThompsonJR, Kimmerer WJ, Feyrer F, Newman K, et al (2010) An analysis of pelagic species decline in the upper San Francisco Estuary using multivariate autoregressive modeling. Ecological Applications 20: 1417–1430. 20666258 10.1890/09-1724.1

[20] Swanson C, Mager RC, Doroshov SI, Cech JJ (1996) Use of salts, anesthetics and polymers to minimize handling and transport mortality in delta smelt. Transactions of the American Fisheries Society 125: 326–329.

[21] Castillo G, Morinaka J, Lindberg J, Fujimura R, Baskerville-Bridges B, et al (2012) Pre-screen loss and fish facility efficiency for delta smelt at the south Delta's State Water Project, California. San Francisco Estuary and Watershed Science 10(4): 1–23.

[22] Feyrer F, Newman K, Nobriga M, Sommer T (2011) Modeling the effects of future outflow on the abiotic habitat of an imperiled estuarine fish. Estuaries and Coasts 34: 120–128.

[23] Feyrer F, Nobriga M, Sommer T (2007) Multi-decadal trends for three declining fish species: habitat patterns and mechanisms in the San Francisco Estuary, California, U.S.A. Canadian Journal of Fisheries and Aquatic Sciences. 64: 723–734.

[24] Newman K (2008) Sample design-based methodology for estimating delta smelt abundance. San Francisco Estuary and Watershed Science. Online serial: http://repositories.cdlib.org/jmie/sfews/vol6/iss3/art3/.

[25] Thomson J, Kimmerer W, Brown L, Newman K, Mac Nally R, et al (2009) Bayesian change-point analysis of temporal patterns in fish abundances in the upper San Francisco Estuary. Ecological Applications 20: 1431–1448. 10.1890/09-0998.1 20666259

[26] Sommer T, Nobriga M, Grimaldo L, Feyrer F, Mejia F (2011) The spawning migration of delta smelt in the upper San Francisco Estuary. San Francisco Estuary and Watershed Science (9)2 http://escholarship.org/uc/item/86m0g5sz.

[27] Dege M, Brown LR (2004) Effect of outflow on spring and summer distribution and abundance of larval and juvenile fishes in the upper San Francisco Estuary. In: Feyrer F, Brown LR, Brown RL, Orsi JJ, editors. Early Life History of Fishes in the San Francisco Estuary and Watershed. American Fisheries Society, Symposium 39, Bethesda, Maryland. 49–65.

[28] Grimaldo L, Sommer T, Van Ark N, Jones G, Holland E, et al (2009) Factors affecting fish entrainment into massive water diversions in a tidal freshwater estuary: can fish losses be managed? North American Journal of Fisheries Management 29: 1253–1270.

[29] Stevens DE, Miller LW (1983) Effects of river flow on abundance of young chinook salmon, American shad, longfin smelt, and delta smelt in the Sacramento-San Joaquin River system. Transactions of the American Fisheries Society 3: 425–437.

[30] Draper AJ, Munevar A, Arora S, Reyes E, Parker N, et al (2004) CalSim: Generalized model for reservoir system analysis. Journal of Water Resource Planning and Management 130: 480–489.

[31] Martin TG, Wintle BA, Rhodes JR, Kuhnert PM, Field SA, et al (2005) Zero tolerance ecology: improving ecological inference by modelling the source of zero observations. Ecology Letters 8: 1235–1246. 21352447 10.1111/j.1461-0248.2005.00826.x

[32] R Development Core Team (2008) R: A language and environment for statistical computing. R Foundation for Statistical Computing, Vienna, Austria. ISBN 3-90005107-0. Available: http://www.R-project.org.

[33] Zeileis A, Kleiber C, Jackman S (2008) Regression models for count data in R. Journal of Statistical Software 27(8). Available: http://www.jstatsoft.org/v27/i08/.

[34] Jackman S (2012) pscl: Classes and methods for R developed in the Political Science Computational Laboratory, Stanford University. Department of Political Science, Stanford University. Stanford, California. R package version 1.04.4. Available: http://pscl.stanford.edu/.

[35] Burnham KP, Anderson DR (2002) Model Selection and Multimodel Inference: A Practical Information-Theoretical Approach (2nd Edition). Springer-Verlag, New York, NY.

[36] Ver Hoef JM, Boveng PL (2007) Quasi-poisson vs. negative binomial regression: how should we model overdispresed count data? Ecology 2766–2772. 10.1890/07-0043.1 18051645

[37] Rockriver A (2004) Vertical distribution of larval delta smelt and striped bass near the confluence of the Sacramento and San Joaquin Rivers. In: Feyrer F, Brown LR, Brown RL, Orsi JJ, editors. Early Life History of Fishes in the San Francisco Estuary and Watershed. American Fisheries Society, Symposium 39, Bethesda, Maryland. 97–108.

[38] Bennett WA, Kimmerer WJ, Burau JR (2002) Plasticity in vertical migration by native and exotic estuarine fishes in a dynamic low-salinity zone. Limnology and Oceanography 47: 1496–1507.

[39] Norcross BL, Shaw RF (1984) Oceanic and estuarine transport of fish eggs and larvae: a review. Transactions of the American Fisheries Society 113: 153–165.

[40] Gibson RN (2003) Go with the flow: tidal migrations in marine animals. Hydrobiologia 503: 153–161.

[41] Hughes DA (1969) Responses to salinity change as a tidal transport mechanism of pink shrimp, *Penaeus duorarum* . The Biological Bulletin 136: 43–53.

[42] Bos AR (1999) Tidal transport of flounder larvae (*Platichthys flesus*) in the Elbe River, Germany. Archive of Fishery and Marine Research 47: 47–60.

[43] Rijnsdorp AD, Van Stralen M, Van Der Veer HW (1985) Selective tidal transport of North Sea plaice larvae *Pleuronectes platessa* in coastal nursery areas. Transactions of the American Fisheries Society 114: 461–470.

[44] Krumme U (2004) Patterns in tidal migration of fish in a Brazilian mangrove channel as revealed by a split-beam echosounder. Fisheries Research 70: 1–15.

[45] Dodson JJ, Dauvin JC, Ingram RG, d’Anglejan B (1989) Abundance of larval rainbow smelt (*Oserus mordax*) in relation to the maximum turbidity zone and associated macroplanktonic fauna of the middle St. Lawrence Estuary. Estuaries 12: 66–81.

[46] Dauvin JC, Dodson JJ (1990) Relationship between feeding incidence and vertical and longitudinal distribution of rainbow smelt larvae (*Osmerus mordax*) in a turbid well-mixed estuary. Marine Ecology Progress Series 60: 1–12.

[47] Laprise R, Dodson JJ (1989) Ontogeny and importance of tidal vertical migrations in the retention of larval smelt *Osmerus mordax* in a well-mixed estuary. Marine Ecology Progress Series 55: 101–111.

[48] Laprise R, Dodson JJ (1989) Ontogentic changes in the longitudinal distribution of two species of larval fish in a turbid well-mixed estuary. Journal of Fish Biology 35(Supplement A)39–47.

[49] Ortner PB, Hill LC, Edgerton HE (1981) In situ silhouette photography of Gulf Stream zooplankton. Deep Sea Research 28: 1569–1576.

[50] Graham DL, Martin DL, Martin JC (2003) *In situ* quantification and analysis of large jellyfish using a novel video profiler. Marine Ecology Progress Series 254: 129–140.

