# Correction: SmeltCam: Underwater Video Codend for Trawled Nets with an Application to the Distribution of the Imperiled Delta Smelt

**DOI:** 10.1371/annotation/0c42ea0f-6d99-44a7-84ff-aeec57133f13

**Published:** 2013-12-13

**Authors:** Frederick Feyrer, Donald Portz, Darren Odum, Ken B. Newman, Ted Sommer, Dave Contreras, Randall Baxter, Steven B. Slater, Deanna Sereno, Erwin Van Nieuwenhuyse

Panel B of Figure 9, "Individual values of delta smelt density (number/10,000 m3) by tide and position in the water column for fish that were (A) found entangled in the mesh of the net when it was retrieved and (B) observed by the SmeltCam." does not appear. Please see the corrected Figure 9 here: http://plosone.org/corrections/pone.0067829.g009.cn.tif

